# Etiology of Dental Anxiety and Dental Phobia: Review

**DOI:** 10.1055/s-0045-1809146

**Published:** 2025-05-22

**Authors:** Agnieszka Piechal, Edyta Siekierska, Kamilla Blecharz-Klin

**Affiliations:** 1 Department of Experimental and Clinical Pharmacology, Medical University of Warsaw, Centre for Preclinical Research and Technology CePT, Warsaw, Poland

**Keywords:** dental anxiety, dental phobia, anxiety disorders, patient anxiety, etiology

## Abstract

This article examines the etiology of dental anxiety, a common yet often overlooked condition in dental practice. Ranging from mild discomfort to severe dental phobia, dental anxiety significantly affects a patient's ability to receive necessary care. Despite its prevalence, it remains underexplored in scientific research, leading to inadequate treatment and insufficient attention. Understanding the factors behind dental anxiety is crucial for prevention and for providing effective psychotherapeutic and pharmacological interventions.

The review was based on a comprehensive search of several scientific databases, including PubMed, Cochrane Database, and Dentistry & Oral Sciences Source. Predefined keywords, such as “Dental Anxiety,” “Dental Phobia,” and “Etiology,” were used to ensure broad coverage of relevant studies. The factors contributing to dental anxiety are multifactorial and involve both internal and external influences. Internal factors, such as genetic predispositions and central nervous system dysfunctions, interact with external influences, including personal experiences, negative past dental encounters, and environmental stimuli. Demographic factors, such as age, gender, and socioeconomic status, along with personal characteristics like temperament and coping mechanisms, further contribute to the development of anxiety.

Previous negative dental experiences and self-reported poor oral health exacerbate anxiety, increasing the likelihood of developing dental phobia, which often results in care avoidance. This avoidance worsens oral health, resulting in conditions such as caries and periodontal disease, and significantly diminishes the quality of life. Addressing dental anxiety is critical for improving patient cooperation and ensuring better long-term oral health outcomes. A more comprehensive understanding of dental anxiety will help identify at-risk patients early, prevent severe forms of dental phobia, and ensure dental care is accessible to all individuals.

In conclusion, dental anxiety is a multifactorial issue that impacts access to dental care and overall oral health. The most significant factor contributing to dental anxiety is trauma associated with previous dental procedures. Expanding the understanding of its causes enables dental professionals to identify at-risk patients and develop tailored interventions. By addressing anxiety early and adopting evidence-based strategies, dental professionals can reduce the prevalence of dental anxiety and improve patient care.

## Introduction

Dental anxiety, which manifests as irrational, intense, and negative emotions associated with visiting the dental office and dental treatment, is a common phenomenon that has been the focus of many researchers for years. 1 A severe form of dental anxiety—dentophobia—is a specific type of phobia recognized by the World Health Organization as a disease entity (ICD-10 in the International Classification of Diseases). High levels of anxiety often lead to neglecting oral health, avoiding contact with the dental office, and frequently cause delays or failures in dental treatment. Depending on the population and measurement techniques, it is estimated that between 2.5 and 20% of individuals experience severe dental anxiety. 2 3 In adults, the global estimated prevalence of dental fear and anxiety is approximately 15.3%. 4 Many studies confirm that fear of the dentist is more common in women and decreases with age. 4 5 6 7 8 9

Negative consequences of dental fear, anxiety, and phobia can include social and psychological effects, such as reduced self-esteem and confidence, feelings of inferiority and shame, or social withdrawal, which arise partly from poor dental health. 10 11 This is due to a tendency to avoid dental care, postpone and cancel scheduled appointments, or not show up at all. 12 13 14 Delaying the resolution of issues is often accompanied by the use of pharmacological aids, such as pain relievers, anti-inflammatory drugs, and antibiotics. This creates a kind of “vicious cycle.” 15 On the other hand, research indicates that dentists often struggle to recognize signs of dental anxiety and dentophobia, as well as the emotional state of patients, which complicates the implementation of appropriate management strategies. 16 17 18 19

Given the scale of the problem and its multidimensional impact on patient functioning, including oral health and dental condition, effective identification of individuals with anxiety disorders and assessment of their severity is necessary. 20 Typically, the initial interaction between the patient and the dentist reveals the first symptoms of dental anxiety or dentophobia, allowing for the development of appropriate treatment strategies. 21

A review of studies relating to the etiology of dental anxiety provides a valuable summary of contemporary knowledge. Dental anxiety is a common phenomenon that is often overlooked in scientific research, causing the problem to grow and remain inadequately addressed, sometimes even ignored by professionals. Understanding all the factors responsible for the development of dental anxiety and dentophobia is essential to prevent this problem in predisposed patients and provide adequate psychotherapeutic and pharmacological treatment.

## Methods

**Fig. 1:**
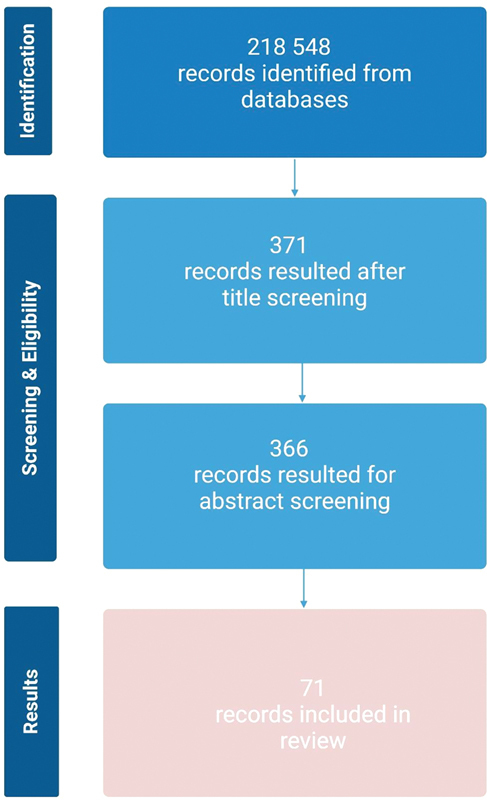
Flowchart of the selection process of the systematic literature review. Created in BioRender. Blecharz-klin, K. (2025) https://BioRender.com/u3m3brz .

This article reviews the literature concerning the etiology of dental anxiety and phobia. To conduct this work, a comprehensive literature search was performed across several databases, including PubMed, Cochrane Database, and Dentistry & Oral Sciences Source. A set of predefined keywords and MeSH (Medical Subject Headings) terms were used to ensure a broad search for relevant studies. The main search terms included: dental anxiety, dental phobia, anxiety disorders, patient anxiety, and etiology. These keywords were chosen based on the primary themes of the review and were refined through initial searches and expert consultation. The search process was conducted by applying Boolean operators (e.g., AND, OR) to combine terms, ensuring that studies addressing both the etiology of dental anxiety and phobia were captured. A three-step process was conducted to select the eligible articles, following the Preferred Reporting Items for Systematic Reviews and Meta-Analyses statement ( Fig. 1 ). 22 Studies were included if they met the following criteria: relevance to the topic, full-text availability, and appropriate study design.

## Results

Initially, 218,548 articles were identified across the databases. After removing duplicates and screening titles and abstracts for relevance, 294 articles were selected for full-text review. The final set of articles included in the review consisted of 71.

A flowchart of the selection process is available in Fig. 1 .

### Causes of Dental Anxiety and Dental Phobia

**Fig. 2:**
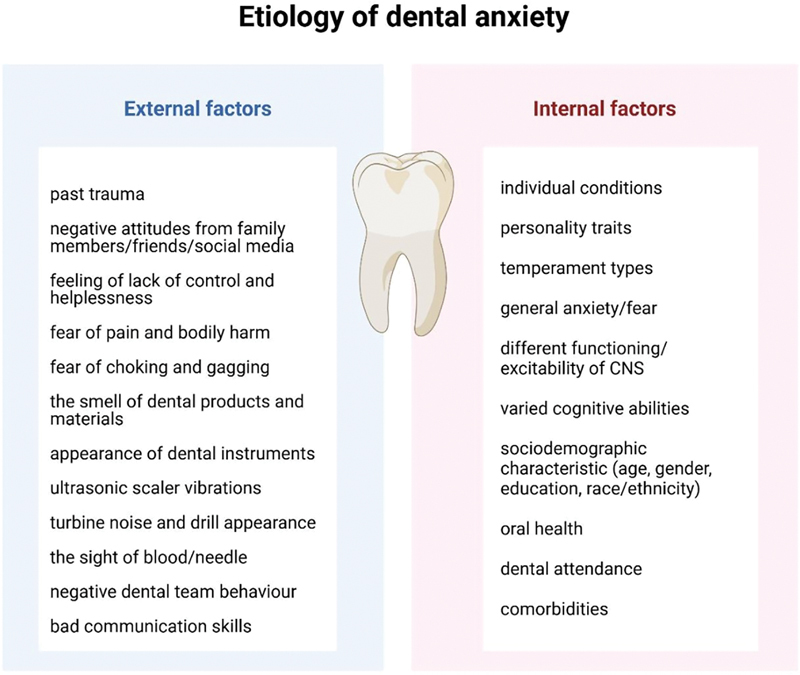
Schematic representation of the etiological factors of dental anxiety, developed based on the literature reviewed. Created in BioRender. Blecharz-klin, K. (2025) https://BioRender.com/y51f388 .

The etiology of anxiety disorders observed in dental patients is complex and multidimensional encompassing a range of external and internal factors ( Fig. 2 ). Factors such as provider attitudes, communication styles, clinical workload, and environmental stressors in the dental practice contribute to anxiety-inducing experiences for patients. Additionally, dental providers may experience their own forms of anxiety or stress, influenced by professional demands, patient expectations, and the pressures to maintain high standards of care. 23 This interconnected dynamic between patients and providers highlights the importance of a more holistic approach that considers both patient and provider well-being in the prevention and management of anxiety in the dental setting.

### External Causes of Anxiety

Dental anxiety and dentophobia consist of several elements: fear of pain, fear of bodily harm, and fear associated with a new, unknown situation.

One of the main external causes of anxiety is the fear of pain related to dental procedures and fear of bodily harm, and fear at the sight of needles and other dental instruments. The specific smells and equipment of the dental office, such as the smell of dental materials, the noise of the dental drill, the vibrations of the ultrasonic scaler, and the sight of blood, can also increase the level of experienced anxiety. 24 25 26 27 28

A direct external cause of dental phobia is often the experience of a traumatic event related to dental or medical treatment. 29 Most patients experiencing anxiety have gone through it in childhood, developing trauma associated with invasive treatments, such as extractions. 3 30 31

Fear of bodily harm includes concerns about injuries to teeth, jawbones, and soft tissues, such as gums and the tongue, during procedures like extractions, mechanical treatment of cavities, or administering local anesthesia. 32 33

Patients who infrequently seek dental care or are undergoing a procedure for the first time often experience anxiety due to fear of the unknown. This anxiety is closely linked to perceptions of the procedure, a lack of awareness, and knowledge about the course of dental procedures or the organization of the visit. 33

A review of the literature also supports that dental anxiety is influenced by external factors like the dentist's behavior and the clinical environment. It is important to highlight the significance of a professional yet empathetic attitude from the dentist and medical staff in preventing such disorders and reducing patient concerns regarding dental treatment. Research indicates that criticizing the patient, negative attitudes from family members—especially parents and peers—toward dental treatment, and the presence of fear in caregivers increase anxiety levels, particularly in young children. 34 35 36

Research highlights that dental professionals play a crucial role in managing dental anxiety, primarily through effective communication, empathy, and creating a calming environment. Studies have shown that patients often experience reduced anxiety when dentists provide clear explanations of procedures, show understanding of patient concerns, and maintain a compassionate attitude. 37 Positive dentist–patient communication is associated with lower levels of anxiety and greater treatment acceptance, as patients are more likely to feel at ease and trust the process. 38 Additionally, dental professionals' ability to address psychological factors, such as fear of pain and past traumatic experiences, can significantly alleviate anxiety. Establishing trust and demonstrating empathy are fundamental to reducing patient stress. 39 Tailoring treatment plans to individual patient needs, combined with anxiety-reducing strategies, is crucial for managing anxiety effectively. 40 Moreover, incorporating psychological strategies such as relaxation techniques and providing a supportive atmosphere can significantly decrease the likelihood of anxiety. 41

Attention should be paid to the prevention of anxiety behavior, especially regarding the earliest dental experiences, which should involve many actions undertaken by both the patient (or the patient's guardian) and the dentists and staff of the dental office ( Fig. 2 ).

From the patient's perspective, several factors are crucial in managing dental anxiety. Early education and the initiation of regular dental care, such as frequent checkups, are essential in reducing anxiety over time. 42 Patients also benefit from care provided at a dental office they trust, under the supervision of a dentist they feel comfortable with. Additionally, it is important for parents, guardians, or household members to avoid making negative comments or discussing dental procedures in a way that might instill fear. A positive attitude toward dental treatments within the household can have a significant impact on the patient's emotional response. 43 Addressing and properly managing any coexisting conditions that may contribute to or exacerbate anxiety is also key to overall well-being. 44 Finally, the use of nonpharmacological and/or pharmacological treatments, as appropriate, can support the patient in managing anxiety during dental visits. 36

From the perspective of the dentist and dental office staff, several key practices are essential in effectively managing dental anxiety. First, minimizing the patient's waiting time for treatment is crucial to reduce stress and anxiety. Additionally, it is important to exhibit high competence, professionalism, and empathy toward patients, as these qualities help build trust and ease patient concerns. Creating comfortable conditions in both the waiting room and the treatment area also plays a significant role in alleviating anxiety. Identifying individuals with dental anxiety early on allows the team to tailor their approach accordingly, and discussing the treatment plan with the patient helps foster a sense of control and transparency. Informing the patient about the course of the procedures and responding appropriately to any signs of discomfort, pain, or anxiety is essential for maintaining trust and ensuring the patient feels supported throughout the process. Effective pain management during and after dental procedures is critical to reduce both physical and psychological distress. Additionally, rewarding patients for demonstrating a positive attitude can reinforce calm and cooperation. Educating patients on methods to cope with dental anxiety, as well as using appropriate pharmacological premedication for those with high levels of anxiety, further supports patient comfort. In cases where oral anxiolytics are not suitable, using nitrous oxide or general anesthesia can provide an alternative means to ensure a more relaxed and stress-free experience.

Preventing the emergence of dental anxiety and gradually reducing its level triggers long-term positive motivation and a good attitude in patients not only toward maintaining oral health but also toward dental procedures, thereby achieving better outcomes in dental treatment.

### Internal Causes of Anxiety

The internal causes of dental anxiety and dentophobia are less understood. They typically include personal characteristics such as age, gender, temperament, different pain sensitivity, individual functioning or excitability of the central nervous system (CNS), and varied cognitive abilities. 45 46 47 48 49 The individual varying intensity of anxiety sensitivity levels serves to lessen or heighten the anxious and fearful response to potentially anxiety-provoking stimuli. 50 Personality and internal vulnerability have a significant impact on the occurrence of anxiety disorders as well as dental anxiety. It can be assumed that dental anxiety may reflect a more general class of anxiety behaviors and is often closely linked to painful stimulus, increased pain perception, as well as a longer lasting memory of the painful experience. For example, higher dental fear occurs in people with alexithymia—an impaired ability to recognize emotions linked with dysfunction of the amygdala a major processing center in mediating many aspects of emotional perception and behavior. 51

Research does not demonstrate clear mechanisms for the inheritance of anxiety; however, there is evidence of genetic predispositions to the occurrence of anxiety. A long-term study by Ray et al involving adolescent identical and fraternal twins indicates that a higher heritability of anxious behaviors is observed in girls than in boys. It has also been shown that there is a greater likelihood of dental anxiety occurring in identical female twins compared to fraternal female twins. 52 53

Analysis of the prevalence of anxiety disorders in identical twins, who are nearly identical in terms of genotype and phenotypic traits, allows for the confirmation of the hypothesis about the correlation of dental anxiety with certain, yet unspecified genetic conditions. 43

Some insights into this subject are provided by research conducted on animals. Zhou et al identified multiple genes in genetically modified animal models that can contribute to the etiology of dental care-related fear and anxiety. 54 The study suggested that genetic loci NTSR1, DMRTA1, and FAM84A may predict greater levels of dental fear.

The mechanisms underlying anxiety at the molecular and neurobiological levels have not been thoroughly investigated. Various elements are involved in the development of anxiety, including membrane receptors, signaling proteins, and transcription factors. It has been shown that the oral microbiome plays a role in mental health due to its involvement in tryptophan metabolism. For example, it has been proven that posttraumatic stress disorder (PTSD) symptoms correlate with a lower abundance of *Haemophilus sputorum* and a higher abundance of *Prevotella histicola* . Simultaneously, relative abundance of *P. histicola* was also positively associated with depressive scores. 55 Tryptophan is an essential amino acid and important precursor for a number of metabolites, for example, kynurenine and the neurotransmitter serotonin. Manipulation of tryptophan levels can modify both peripheral and central serotonin levels. The structure and composition of the salivary microbiome periodontal as well as tryptophan metabolism in the oral–brain axis—a bidirectional system between the brain and gastrointestinal tract—significantly affect mental health outcomes. 55

During the onset of dental anxiety, stressful stimuli, such as the sound of dental instruments, the sight of dental tools, and the anticipation of pain, initiate a sequence of neural responses that lead to the experience of fear. This process involves a coordinated activation of several brain structures that are critical for sensory processing, emotional response, and fear regulation.

**Fig. 3:**
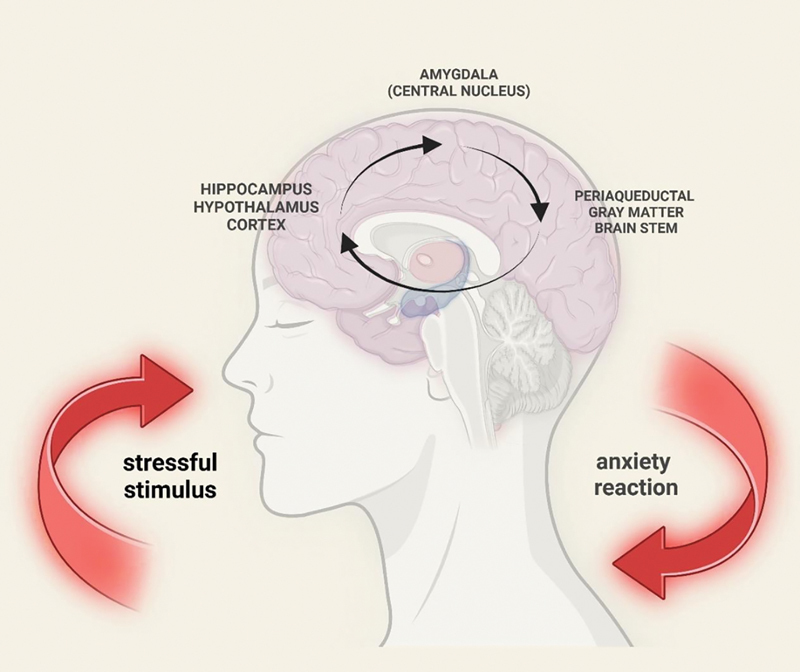
The impact of stressful stimuli on brain responses and the manifestation of dental anxiety. The figure illustrates the cascade of interactions between various brain structures triggered by a stressful stimulus (e.g., dental-related stimuli). A stressful stimulus activates the hippocampus and hypothalamus, which play key roles in processing emotional responses and regulating stress reactions. This activation leads to engagement of the cortex, which is responsible for evaluating and interpreting the situation as a potential threat. Subsequently, the periaqueductal gray matter and brainstem are activated, initiating an anxiety response. This process results in physiological manifestations of anxiety, such as increased heart rate, muscle tension, and other bodily responses associated with the stress reaction. Created in BioRender. Blecharz-klin, K. (2025) https://BioRender.com/m05p210 .

The process begins when sensory stimuli are perceived by the sensory systems and processed by the thalamus, which serves as a relay station for sensory information. From the thalamus, the sensory signals are sent to higher brain areas, including the amygdala. It is believed that the key center for processing stimuli and coordinating anxiety responses is the amygdala located in the temporal lobe, which receives threat information from the cortex, hippocampus, and thalamus. This structure of the CNS is a “hub” for emotional memory processing, determining the “anxiogenic” nature of situations and sending decisions to effector centers: the periaqueductal gray matter, hypothalamus, and brainstem. These structures, in turn, trigger physiological, behavioral, and hormonal responses that condition the emergence of anxiety ( Fig. 3 ). 56 57 The amygdala activates the autonomic nervous system, preparing the body for a “fight or flight” reaction. This includes increasing heart rate, heightened alertness, and other physiological changes associated with anxiety. 58 As the emotional response is initiated by the amygdala, the prefrontal cortex (PFC) attempts to regulate this reaction. The PFC is responsible for higher-order cognitive functions, including decision-making and emotional regulation. In individuals with dental anxiety, the PFC may struggle to effectively inhibit the heightened fear response initiated by the amygdala, leading to failures in emotional regulation and the persistence of anxiety. 59 The hippocampus is involved in memory processing and emotional regulation, which, in this case, may trigger recollections of past stressful dental experiences. The periaqueductal gray matter and brainstem are responsible for activating the physiological components of the anxiety response, including autonomic functions like heart rate acceleration and muscle tension.

Overall, the experience of dental anxiety is a result of the interaction between sensory stimuli and these brain structures.

Areas of the brain involved in the formation of anxiety responses have the ability to communicate with each other through neurohormones and neurotransmitters. Dysregulation in any of these brain areas can result in heightened or sustained anxiety, commonly observed in individuals with dental phobia. Studies have shown a relationship between certain neuropeptides and the occurrence of anxiety and depression. Neuropeptides, small polypeptide molecules, are synthesized and released by neurons. Among them, neuropeptides such as oxytocin, vasopressin, corticotropin, corticotropin-releasing hormone, neurotensin, and cholecystokinin play a role in the formation of anxiety and depression responses ( Table 1 ). 60

**Table 1:** Characteristics of selected neuropeptides with potential influence on anxiety and depression

Neuropeptide	Brief description
Oxytocin	A neuropeptide synthesized in the hypothalamus involved in memory processes, modification of social and anxiety-related behaviors. Released by the central nucleus of the amygdala, it directly influences GABAergic system activation and reduces anxiety responses
Vasopressin	A hormone synthesized by the hypothalamus and released by the posterior pituitary. It regulates kidney function and blood vessels. Vasopressin likely interacts with oxytocin to regulate the response to stress stimuli. High levels of vasopressin can induce anxiety responses and also affect the serotoninergic system
Corticotropin	A hormone secreted by the pituitary gland that stimulates the production of glucocorticoids in the adrenal cortex. In response to stress stimuli, the level of corticotropin in the blood increases
Corticotropin-releasing hormone (CRH)	A neurotransmitter released from the hypothalamus and the central nucleus of the amygdala, inducing the secretion of corticotropin by the pituitary gland. It is considered the main mediator of stress, triggering or enhancing anxiety behaviors. It also affects appetite, sleep, learning, and proinflammatory processes
Neurotensin	A neuropeptide associated with the brain–gut axis, activated during the digestion of fats and proteins. Its role in the mechanism of anxiety formation is not fully understood, but it is believed that high levels of neurotensin combined with high levels of corticotropin-releasing hormone increase the likelihood of anxiety behaviors
Cholecystokinin	A neuropeptide linked to the brain–gut axis, mainly released in the duodenum and jejunum. It stimulates the pancreas and gallbladder. Decreased levels of cholecystokinin promote anxiety responses

It is important to note the impact of stress on amygdala activity. In response to a stressor, such as dental visits, there are changes in neuronal function and hyperactivity of the amygdala. As a result of prolonged stress exposure, many proinflammatory cytokines are released by the immune system cells. This creates an inflammatory state in the nervous system, which is a significant pathophysiological factor contributing to neuropsychiatric disorders, including anxiety behaviors. Neuroimaging studies indicate that amygdala activity is increased in individuals with autoimmune diseases, such as multiple sclerosis and rheumatoid arthritis. 56

Moreover, in individuals with autism spectrum disorder, there is variability in the maturation of the amygdala, with hypertrophy of the amygdala nuclei during infancy and early childhood, and a reduction in volume of these structures in adults. These changes correlate with the severity of cognitive and social function disorders as well as anxiety behaviors. 61 62

Research also shows that a significant role in the development of anxiety behaviors is played by a deficiency in GABAergic transmission and abnormalities in serotoninergic transmission. 63 This explains the high effectiveness of benzodiazepines, which modulate GABAergic transmission, and antidepressants that affect serotonin reuptake in reducing dental anxiety and treating dentophobia. The GABAergic system includes GABA membrane receptors, which bind gamma-aminobutyric acid, the main inhibitory neurotransmitter. Among the three identified receptor types (A, B, and C), the GABA _A_ receptor is key in the mechanism of anxiety formation. The release of gamma-aminobutyric acid from the central nucleus of the amygdala and activation of the GABA _A_ receptor decreases neuronal excitability in the hypothalamus and inhibits CNS activity. A deficiency in GABAergic transmission results in increased neuronal excitability and enhanced nerve impulse conduction in the brain, which may be related to anxiogenic effects, meaning it induces anxiety disorders. 63

Additionally, literature analyses have shown that sensory (afferent) neurons of the vagus nerve, which belong to the brain–gut axis, also mediate anxiety. Nerve impulses from the gastrointestinal tract are transmitted by afferent neurons to brain structures responsible for coordinating anxiety responses, including the amygdala. Thus, stimuli from the vagus nerve influence GABAergic transmission, and a reduction in nerve impulse flow from the vagus nerve, such as due to injury, results in increased GABAergic transmission. 63

Furthermore, patients suffering from dental anxiety have an increased risk of coexisting various psychiatric disorders, with a tendency that the higher the level of anxiety, the greater the likelihood of occurrence or exacerbation of existing abnormalities.

Scientific studies suggest that dental anxiety may be associated with the cooccurrence of various other health issues in patients. For example, conditions such as molar-incisor hypomineralization have been found to correlate with dental anxiety. 64 Furthermore, several neuropsychiatric problems may also be linked to heightened levels of dental anxiety. These include obsessive-compulsive disorders, which can manifest as avoidance of dental visits due to an excessive fear of infections and microorganisms. Social phobia, characterized by a fear of judgment by others, is another anxiety-related issue that can impact dental visits. Additionally, agoraphobia, which involves a fear of being outside the home, especially in public spaces, may hinder individuals from seeking necessary dental care. Other conditions, such as depression and mood disorders, as well as PTSD, have also been identified as factors that may exacerbate dental anxiety. Moreover, the abuse of alcohol and/or psychoactive substances has been observed in some individuals as a coping mechanism for managing the distress related to dental visits. 65 66 67 These associations highlight the complex interplay between dental anxiety and various physical and psychological health issues, underscoring the need for a comprehensive approach to patient care.

Anxiety is also a common component of health issues not directly related to CNS function, such as myocardial infarction, pulmonary embolism, or endocrine disorders.

## Methods of Measuring Dental Anxiety

Before effective dental treatment can commence for a patient with anxiety, assessing the severity of that anxiety is essential. Based on this assessment, an appropriate treatment plan can be established, incorporating nonpharmacological methods to reduce anxiety.

Methods for assessing dental anxiety are broadly categorized into physiological, behavioral, and psychological approaches, each offering unique insights into the patient's experience. Physiological methods involve measuring bodily responses that indicate anxiety during dental visits, such as elevated blood pressure, increased heart rate, muscle tension, hand sweating, or stress markers in saliva or serum. 68 These measures provide objective data on the body's stress response, but they may not fully capture the psychological aspects of anxiety or the patient's subjective experience.

Behavioral approaches, on the other hand, focus on observing actions or behaviors that are indicative of anxiety, such as avoidance of appointments, fidgeting, or verbal expressions of fear. These approaches can help identify anxious behaviors in patients who may not express their anxiety through physiological markers, but they also rely on the observer's interpretation, which can introduce subjectivity. Behavioral methods, primarily used with children, involve observing the patient's behavior and having the clinician mark the corresponding behavior category on a behavioral scale.

Finally, psychological approaches involve self-reported questionnaires or scales that assess the emotional and cognitive components of dental anxiety. Psychological methods for assessing dental anxiety often rely on patient-completed questionnaires or scales that provide valuable insights into a patient's level of anxiety regarding dental treatments. A widely used tool is the Corah's Dental Anxiety Scale (DAS), consisting of four questions related to common dental situations. 69 This scale is simple and quick to administer, making it a popular choice in clinical settings. However, a notable limitation of the DAS is that it does not include a question about local anesthesia, which is a common source of anxiety for many patients, particularly in relation to injections.

To address this gap, the Modified DAS (MDAS) was developed by adding a fifth item specifically about local anesthesia. 70 This modification makes the MDAS a more comprehensive tool for assessing dental anxiety, capturing concerns related to injections, which are often a significant trigger of fear. Despite this improvement, the MDAS remains relatively general, and like the DAS, it may not fully capture the multifaceted nature of dental anxiety in all individuals.

The Kleinknecht's Dental Fear Scale (DFS) is another widely used instrument, including 20 items focusing on various aspects of dental visits, such as making an appointment or hearing the sound of a dental drill. 71 These items are evaluated on a 5-point Likert scale. Factor analysis of the DFS has identified three key parameters: avoidance of dental treatment, somatic symptoms of anxiety, and anxiety triggered by dental treatment-related stimuli. While the DFS provides a more nuanced understanding of dental anxiety by categorizing these different aspects, its length and detailed nature may make it more time-consuming for patients, which could limit its utility in fast-paced clinical settings.

Lastly, Gatchel's 10-Point Dental Fear Scale takes a different approach by using a single item where participants rate their anxiety on a scale from 1 to 10. A score of 1 represents no anxiety, while a score of 8 or above indicates significant anxiety. Although this scale is very simple and quick to use, its one-dimensional nature can be limiting, as it does not capture the diverse factors that may contribute to dental anxiety, such as specific procedural fears or psychological traits that might underlie anxiety.

In conclusion, while these scales provide useful tools for assessing dental anxiety, each has its strengths and limitations. The DAS and MDAS are relatively simple and easy to use, but they may not fully encompass the various triggers of dental anxiety. The DFS offers a more detailed assessment but may be too complex for some clinical situations. Gatchel's 10-Point Scale is very efficient, yet it lacks depth in its assessment of anxiety's underlying causes. Together, these scales contribute to a better understanding of dental anxiety, though there is still a need for more comprehensive tools that can capture the full range of patient experiences.

## Discussion

Dental anxiety is a significant factor contributing to the phenomenon of patients avoiding treatment. Feelings of anxiety and the strong negative emotions associated with dental treatment affect patients of all ages—both children and adults. 72 Dental anxiety and dentophobia not only complicate dental treatment due to avoidance behaviors and hindered cooperation but also diminish the patient's quality of life. These disorders are significant risk factors for developing cavities and periodontal disease. 73 74 The primary factors responsible for the emergence of anxious behaviors, in addition to personal traits, are unpleasant experiences related to dental treatment, which can intensify and become entrenched in response to repeated stressors or comorbid conditions. In particular, these studies consistently highlight early negative experiences and trauma as the primary initiating factor of dental anxiety, emphasizing it as a significant issue. This issue was identified by all papers included in the review.

It is the role of the dentist to recognize anxious patients and to prevent the development of negative patterns of anxious behavior through a proper attitude, professional and empathetic approach, as well as preventive actions, particularly concerning early dental experiences. 48 The importance of a dentist in anxiety management is critical, yet often underemphasized in the literature. A dentist plays a role not only in diagnosing dental anxiety but also in implementing strategies that can significantly alleviate it. To improve the practical utility of this review, it is essential to highlight specific tactics that can be employed by dental professionals. Behavioral approaches such as cognitive-behavioral therapy techniques, relaxation exercises, and guided imagery can be used during dental appointments to help patients manage anxiety. Additionally, pharmaceutical interventions, including the use of anxiolytics or sedatives like benzodiazepines or nitrous oxide, can be considered for patients with high levels of anxiety. 75 Environmental adjustments in the dental office, such as a calming atmosphere with soothing music, comfortable seating, or even the option of a quiet room, can create a less stressful environment and contribute to anxiety reduction. By incorporating these specific strategies, dentists can play a more proactive and effective role in managing dental anxiety, improving the overall patient experience, and encouraging regular dental visits. 30

Furthermore, it is necessary to establish educational programs in this area, as increasing awareness among dentists and patients experiencing dental anxiety will enable better cooperation in oral health, creating opportunities to break the “vicious cycle” of dental anxiety and treatment avoidance.

This review provides a comprehensive examination of the various factors contributing to dental anxiety and phobia, shedding light on the complex interplay between psychological, physiological, and environmental influences. By synthesizing current research, this review contributes to a deeper understanding of the etiology of these conditions, highlighting the need for tailored approaches to treatment and prevention. The findings are particularly valuable for health care providers, as they can inform strategies for identifying and addressing dental anxiety early in the patient care process. Dentists, hygienists, and mental health professionals will benefit from a more nuanced understanding of the factors contributing to dental phobia, enabling them to offer more empathetic and effective care. Patients stand to gain from improved treatment experiences and outcomes, as practitioners adopt evidence-based approaches to managing anxiety. Additionally, policymakers and public health officials can use the insights from this review to advocate for and implement better mental health support systems within dental care settings, ensuring that patient anxiety is recognized as a significant barrier to health care access and treatment.

*Strengths and Limitations* : This review's strengths lie in its broad, multidisciplinary approach, integrating insights from psychology, dentistry, and public health. By considering a wide range of factors, from cognitive and emotional triggers to sociocultural influences, the review offers a holistic understanding of dental anxiety and phobia. However, a limitation of this review is the reliance on existing literature, which may present biases or inconsistencies in methodology, as not all studies employ standardized or universally accepted measures of dental anxiety. Additionally, while the review explores several factors, the heterogeneity of anxiety experiences among different populations means that some patient groups may be underrepresented. Future research should aim to fill these gaps by exploring dental anxiety in diverse demographic and cultural contexts.

## Conclusion

In conclusion, dental anxiety is a multifaceted condition with complex etiological factors that include psychological, biological, and environmental influences. The reviewed studies highlight the significant role of early experiences, particularly negative or traumatic dental encounters, in shaping anxiety levels. Furthermore, psychological factors such as preexisting mental health conditions, including generalized anxiety, social phobia, and depression, are commonly observed in individuals with dental anxiety. Physiological factors, such as genetic predisposition and heightened sensitivity to pain, also contribute to the development and severity of dental phobia. Environmental factors, including the behavior of dental providers and familial attitudes toward dental care, play a critical role in either mitigating or exacerbating anxiety. Key recommendations for future research include the need for more longitudinal studies to better understand how dental anxiety develops over time and the exploration of specific interventions that target both the psychological and physiological aspects of anxiety. Additionally, a deeper examination of the diverse cultural and demographic factors influencing dental anxiety will enhance the ability to offer personalized care to patients from varied backgrounds. In practice, it is crucial for dental providers to adopt a more holistic approach in treating anxious patients, integrating psychological support and anxiety-reducing techniques into dental care. Establishing a patient-centered care model that considers both the emotional and physical aspects of anxiety can improve patient compliance and overall treatment outcomes.

By continuing to advance our understanding of the etiology of dental anxiety, future research and practice can reduce the burden of this condition, ensuring that patients experience more comfortable and effective dental care.
